# Reduction of focal sweating by lipid nanoparticle-delivered myricetin

**DOI:** 10.1038/s41598-020-69985-x

**Published:** 2020-08-04

**Authors:** Choongjin Ban, Joon-Bum Park, Sora Cho, Hye Rin Kim, Yong Joon Kim, Young Jin Choi, Woo-Jae Chung, Dae-Hyuk Kweon

**Affiliations:** 1 Department of Environmental Horticulture, University of Seoul, 163 Seoulsiripdaero, Dongdaemun-gu, Seoul, 02504 Republic of Korea; 2 Department of Integrative Biotechnology, Sungkyunkwan University, 2066 Seoburo, Suwon, Gyeonggi 16419 Republic of Korea; 3 Interdisciplinary Program in BioCosmetics, Sungkyunkwan University, 2066 Seoburo, Suwon, Gyeonggi 16419 Republic of Korea; 4 Institute of Biomolecule Control, Sungkyunkwan University, 2066 Seoburo, Suwon, Gyeonggi 16419 Republic of Korea; 5 Biologics Research Center, Sungkyunkwan University, 2066 Seoburo, Suwon, Gyeonggi 16419 Republic of Korea; 6 Department of Agricultural Biotechnology, Seoul National University, 1 Gwanakro, Gwanakgu, Seoul, 08826 Republic of Korea; 7 Center for Food and Bioconvergence, Seoul National University, 1 Gwanakro, Gwanakgu, Seoul, 08826 Republic of Korea; 8 Research Institute of Agriculture and Life Sciences, Seoul National University, 1 Gwanakro, Gwanakgu, Seoul, 08826 Republic of Korea

**Keywords:** Neurochemistry, Nanoparticles, Drug delivery, Nanoparticles, Drug delivery, Neuromuscular junction, Inhibition, Neurotransmitters, Drug delivery, Nanoparticles, Drug delivery, Colloids

## Abstract

Myricetin—a flavonoid capable of inhibiting the SNARE complex formation in neurons—reduces focal sweating after skin-application when delivers as encapsulated in lipid nanoparticles (M-LNPs). The stability of M-LNP enables efficient delivery of myricetin to sudomotor nerves located underneath sweat glands through transappendageal pathways while free myricetin just remained on the skin. Furthermore, release of myricetin from M-LNP is accelerated through lipase-/esterase-induced lipolysis in the skin-appendages, enabling uptake of myricetin by the surrounding cells. The amount of sweat is reduced by 55% after application of M-LNP (0.8 mg kg^−1^) on the mouse footpad. This is comparable to that of subcutaneously injected anticholinergic agents [0.25 mg kg^−1^ glycopyrrolate; 0.8 U kg^−1^ botulinum neurotoxin-A-type (BoNT/A)]. M-LNP neither shows a distal effect after skin-application nor induced cellular/ocular toxicity. In conclusion, M-LNP is an efficient skin-applicable antiperspirant. SNARE-inhibitory small molecules with suitable delivery systems have the potential to replace many BoNT/A interventions for which self-applications are preferred.

## Introduction

Focal hyperhidrosis is an idiopathic abnormality characterized by excessive sweating concentrated in certain regions of the body, such as palms, soles, face, and armpits^1^. Nearly 3% of the general population—most people aged 25‒64 years—experiences hyperhidrosis. Due to its interference with daily activities, this condition carries a substantial emotional, psychological, social, and professional burden^2^, even resulting in social anxiety disorders^3^. Various treatments for blocking the sweating-mechanisms have been employed to treat this abnormality. Systemic agonists, antiperspirants, iontophoresis, local excision, and botulinum neurotoxins type A (BoNT/A) have been approved by the US Food and Drug Administration^4^. Systemic agonists are usually used to treat generalized hyperhidrosis. Glycopyrrolate, phentolamine, clonidine, propranolol, and diltiazem lessen the symptoms as they act as anticholinergic^5^, α-adrenergic^6^, α2-adrenergic^7^, β-blocker^1^, and calcium channel blockers^8^, respectively. These agents show various side effects such as daytime sedation, dry mouth, constipation, blurred vision, urinary retention, and tachycardia^1^. Aluminum chloride solutions mechanically obstruct sweat gland ducts, which in turn leads to atrophy of the eccrine acini^9^. Limitations of aluminum chloride include skin irritation, burning, stabbing dysesthesias, temporary relief, and cumbersome application^4^. Iontophoresis with tap water or glycopyrrolate solution also has drawbacks, such as erythema, local burning pain, and blistering^10^. The adverse effects of surgical treatments include scars, paresthesia, pigmentation, partial alopecia, compensatory hyperhidrosis, Horner’s syndrome, hemothorax, and pneumothorax despite improvement in the symptoms^1^.

BoNT/A, produced by an anaerobic bacterium *Clostridium botulinum*, is a safe and effective method for treating focal hyperhidrosis, providing long-lasting results without invasive surgical procedures. When BoNT/A is intradermally injected, it blocks the exocytotic release of acetylcholine from the sudomotor synapses^11^. BoNT/A is transported into sudomotor neurons by binding to proteins on the presynaptic membrane. After endocytosis, the light chain of BoNT/A is released into the cytosol through the endosomal membrane. The light chain proteolytically cleaves the synaptosome-associated protein 25 kDa (SNAP25), thereby preventing the formation of the soluble *N*-ethylmaleimide-sensitive factor attachment protein receptor (SNARE) complex. Because SNARE complex formation is essential for opening the fusion pore through which neurotransmitters are released, the cleaved SNAP25 fails to mediate the membrane fusion involved in acetylcholine release^12^. Due to effectiveness and as a result of high level of patient satisfaction, many recent studies for treating focal hyperhidrosis have focused on the use of BoNTs^13–15^. Nevertheless, BoNT/A is still a clinical strategy with drawbacks of injection pain, high costs, paralysis, and compensatory hyperhidrosis^1,4,16^. Most importantly, BoNT/A cannot be made as self-applicable cream-type drug but needs to be injected by a doctor in a hospital environment because it is a fatal neurotoxin and because macromolecules with a molecular weight of 150–950 kDa cannot penetrate to the sudomotor nerves via skin application.

We have previously shown that myricetin, a plant flavonoid with molecular weight of 318 Da, interferes with neuronal SNARE complex formation^17–21^. When myricetin is present, SNARE complex formation is allowed only in the *N*-terminal half whereas *C*-terminal half zippering is inhibited by myricetin. Membrane fusion mediated by SNARE proteins is arrested at the hemifusion stage by myricetin. This in turn leads to inhibition of neurotransmitter release from the neuron and flaccid paralysis of muscles. Thus, myricetin blocks neuroexocytosis by inhibiting SNARE complex formation, whereas BoNT do this by proteolyzing SNARE proteins. Myricetin is a flavonol commonly present in vegetables, fruits, nuts, berries, tea, and wine^22^. The safety profiles of flavonoids are well-known^22^, suggesting that myricetin is a safe BoNT-like compound derived from plants. In the present study, we ask whether myricetin applied on skin reduces sweating similar to injected BoNT/A.

Several delivery systems are known to enhance the absorption and bioavailability of dermally applied hydrophobic bioactives^23^, however, the delivery system targeting sudomotor nerves should be able to travel along the sweat ducts. Furthermore, the delivery system needs to release drugs in the deep bottom of the glands. Here, lipid nanoparticles (LNP) were recruited to effectively deliver myricetin to the sudomotor nerve located underneath sweat glands. The present study demonstrates that myricetin-loaded lipid nanoparticles (M-LNPs), which can be disassembled in the transappendageal pathway, efficiently reduce sweating by delivering myricetin deeply into the ducts.

## Results and discussion

### Characteristics of lipid nanoparticles

**Figure 1:**
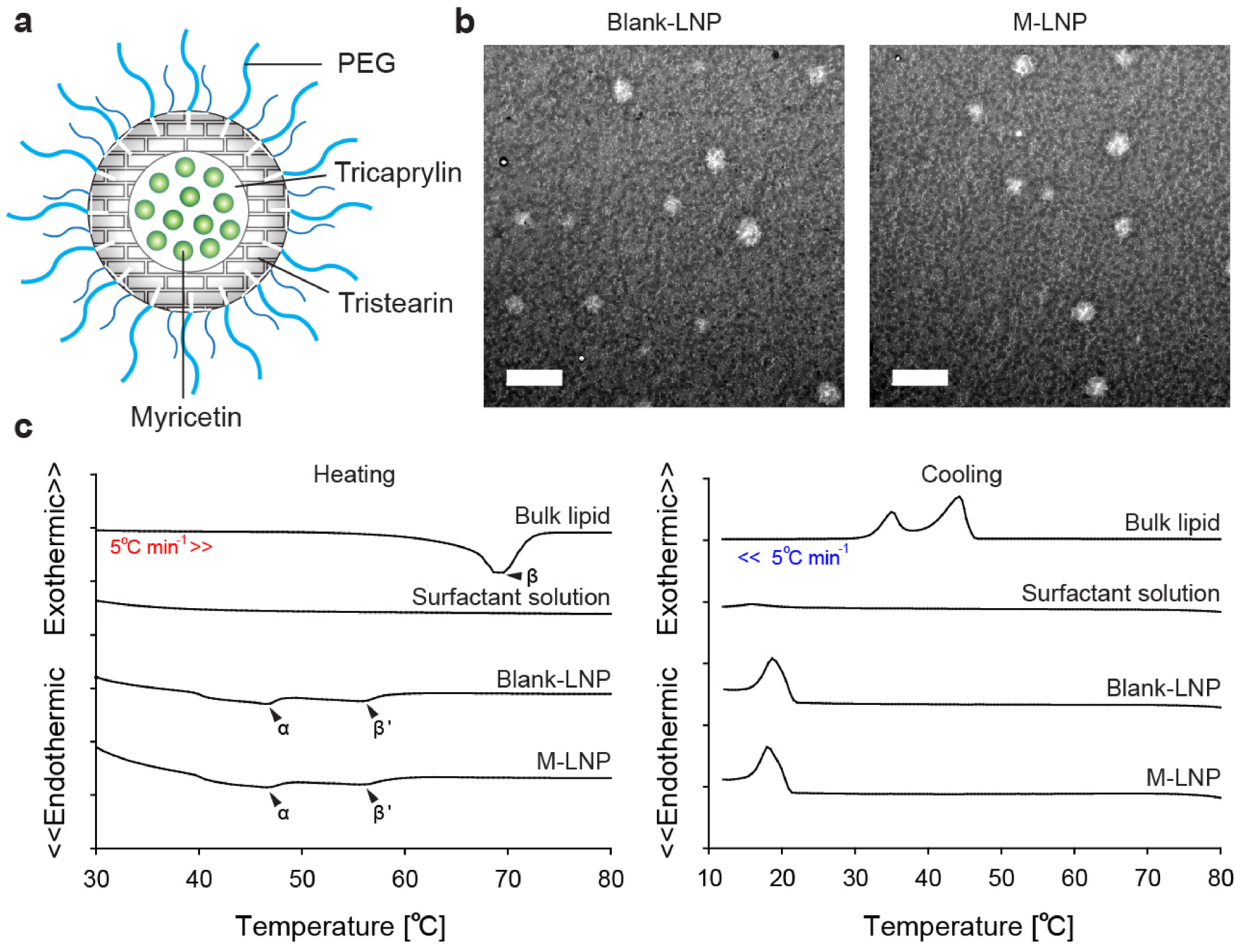
Characterization of LNPs. (**a**) Cartoon representation of the M-LNP. (**b**) Micro-morphologies of blank-LNP and M-LNP obtained by transmission electron microscopy (scale bars, 1 μm). (**c**) Heating- and cooling-calorigrams of bulk lipid (70 wt% tristearin + 30 wt% tricaprylin), surfactant aqueous solution (7.5 wt% Brij S100 + 7.5 wt% Pluronic P123), Blank-LNP, and M-LNP obtained with differential scanning calorimeter at a rate of 5 °C min^−1^.

The delivery system targeting sudomotor nerves needs to be stable in aqueous solution but drug-releasable in response to a certain environment of the transappendageal pathway to efficiently deliver myricetin deeply into the sweat glands. M-LNP was assembled to protect and solubilize myricetin using tristearin (TS) and tricaprylin (TC) comprising the lipid matrix (Fig. 1a). After M-LNP and Nile red- (NR-) LNP containing myricetin and NR, respectively, were prepared using fats (TS), oils (TC), and surfactants [Brij S 100 (BR) and Pluronic P-123 (PL)], their physicochemical properties, including yield, mean particle size (PS), ζ-potential (ZP), and surfactant surface load (Γ_s_) were determined (Supplementary Table S1). PEGylated surfactants covering the surface of LNPs sterically hinder shear-induced particle aggregation. Especially, the lipophilic part (C18 chain) of BR facilitates the surface-initiated crystallization of TS and shields the core of LNPs^24^. Yields of the LNP preparations were > 90% and most myricetin added to form M-LNP was encapsulated in the lipid matrix with an entrapment efficiency (EE) of ~ 99%. PS values of LNPs were below 200 nm, indicating good colloidal stability. The LNPs showed high Γ_s_ values of 28‒30 mg surfactants per m^2^ surface. The surface charge (ZP) of LNPs was almost zero (− 4.6 to − 2.5 mV) because possible negatively charged impurities such as free fatty acids (FFAs) were masked by the high covering effect of PEGylated surfactants present at the lipid-water interface^25^. Unstable particles and aggregates of LNPs usually show rod-, needle-, or platelet-like shapes due to the polymorphic transition of lipid matrices from the α- or β'-form to β-form^26,27^. However, transmission electronic microscopic (TEM) images showed that LNPs retained a spherical shape, and their size was in accordance with the sizes of 170‒184 nm obtained by dynamic light scattering measurements (Fig. 1b, Supplementary Table S1).

Differential scanning calorimetric (DSC) results were obtained to determine thermal properties of LNPs or of each LNP component (Fig. 1c). As shown in heating- and cooling-calorigrams for aqueous surfactant solutions, surfactants alone without lipids neither melted nor crystallized in the aqueous condition, yielding no change in the calorigrams. The endothermic peak representing melting of β-form crystals of TS appeared at ~ 70 °C in the heating-calorigram of the bulk lipid. Endothermic peaks in the heating-calorigrams of LNPs were attributed to the polymorphism of lipids, where peaks at ~ 47 °C and ~ 56 °C represented the melting of α- and β'-form crystals of TS, respectively. These two peaks typically represent TS melting at the core and outer layer of LNPs which is densely anchored to the high-melting surfactant at the interface. The lowered transition temperature compared with bulk lipid was due to more disordered α- and β'-form crystals of the LNPs than the β-form crystals of the bulk lipid, resulting in the spherical shape of LNPs. While exothermic peaks of bulk lipid were observed at ~ 44 °C and ~ 35 °C in cooling-calorigrams, the peaks of LNPs appeared at ~ 18 °C, which were attributed to crystal imperfections due to the limited space in nanoscale lipid particles. The lowered crystallization temperature compared with bulk lipid indicates the absence of large particles and destabilized LNPs in the dispersion^28^. These results suggest that M-LNP had very good colloidal stability, thus preventing LNP coalescence, flocculation, and aggregation.

### Sweat reduction by myricetin-loaded lipid nanoparticles

**Figure 2:**
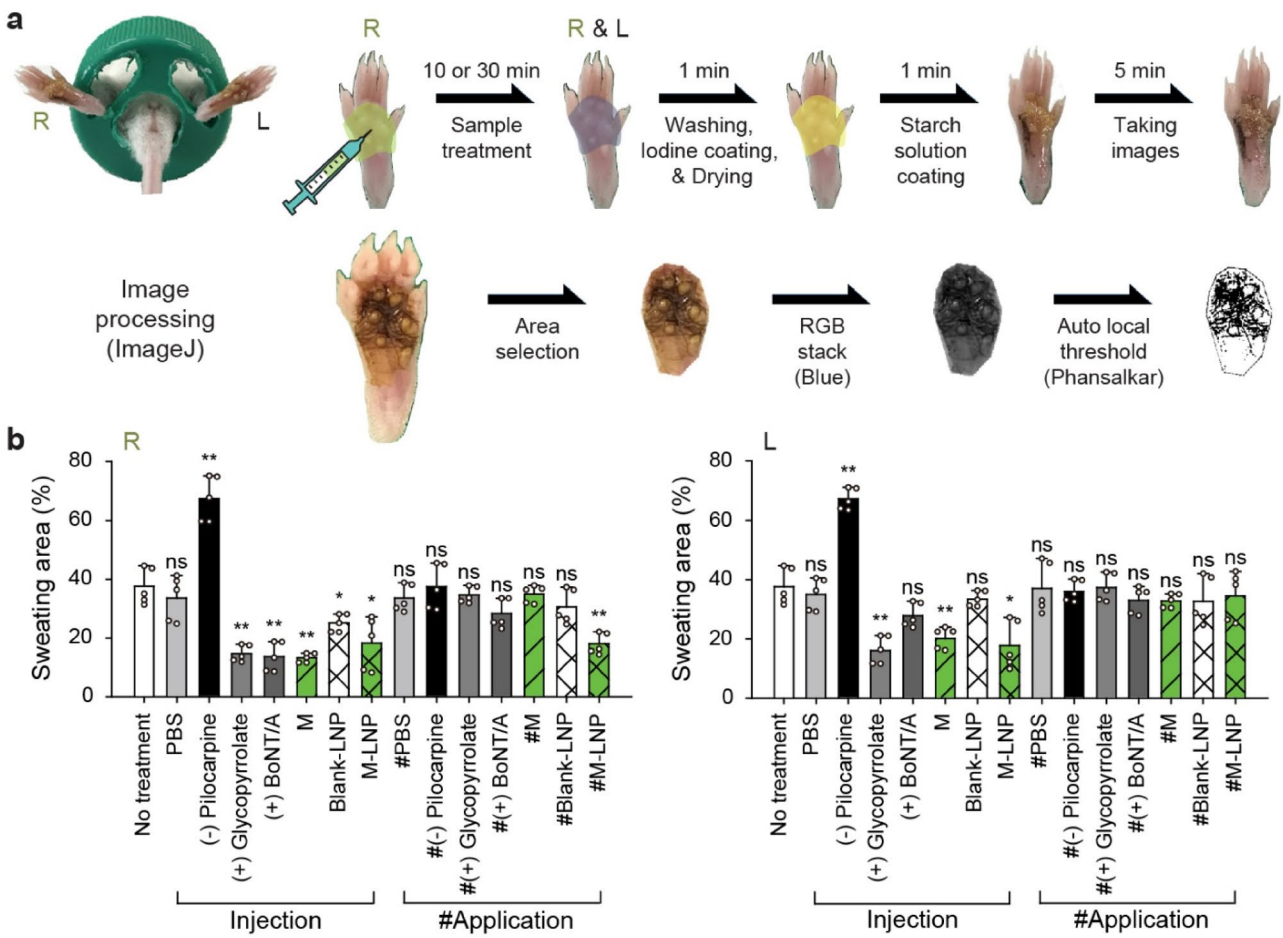
Sweat reduction by M-LNP. (**a**) Procedure of sweat assay on mouse footpads using the iodine/starch method. (**b**) Sweating area (%Area of black pixel fraction) on the right (R) and left (L) hind footpads of mouse groups treated with agents only on the right footpads by subcutaneous injection or skin-application. Groups: no treatment, PBS (0.8 mL kg^−1^), pilocarpine HCl (2.5 mg kg^−1^, negative control), glycopyrrolate bromide (0.25 mg kg^−1^, positive control), BoNT/A (Meditoxin; 0.8 U kg^−1^, positive control), myricetin (M; 0.8 mg kg^−1^), Blank-LNP (0.8 mL kg^−1^), and M-LNP (0.8 mg kg^−1^). Data are expressed as the mean ± s.d. (standard deviation; *n* = 5; Student’s *t*-test; *ns* non-significant, *p < 0.05, **p < 0.01).

Groups of mice were treated with phosphate buffered saline (PBS), pilocarpine, glycopyrrolate, BoNT/A, myricetin solution, Blank-LNP or M-LNP. Pilocarpine is a cholinergic agonist used to stimulate the secretion of large amounts of saliva and sweat^29^. Glycopyrrolate is an anticholinergic agent with primarily anti-muscarinic effects that is known to decrease sweat^30^. Seven groups of mice were subcutaneously (SC) injected on the right hind footpads with the agents and the same agents were directly skin-applied to the right hind footpads of the other seven groups of mice. After 10 and 30 min of SC-injection and application, respectively, the amount of sweat from both right and left footpads was determined using the iodine/starch method (Fig. 2a, Supplementary Fig. S1).

PBS-treated groups showed a 34‒37% sweating area from the iodine/starch-based sweat assay (Fig. 2b). This value was regardless of the treatment method and was similar to the non-treated group (~ 38%). Both right and left footpads showed the same sweating area after PBS injection or skin-application. In contrast, SC-injected pilocarpine and glycopyrrolate, a sweat-secretion enhancer and reducer, respectively, dramatically affected the sweating area. When pilocarpine was injected, the sweating area was increased by ~ 2-times whereas glycopyrrolate injection lowered the sweating area to as low as 15%. Interestingly, sweating from the left footpad was also affected by SC-injection of pilocarpine and glycopyrrolate on the right footpad indicative of systemic effects. However, skin-applied pilocarpine and glycopyrrolate did not show any significant effect on the sweating of any footpad. SC-injection of BoNT/A reduced the sweating area on only the right hind footpads to 14%. It is thus likely that its large molecular weight and binding specificity to presynaptic neuronal membrane near the site of injection limited the distal effect of BoNT/A^12^. Skin-application of BoNT/A was not effective in reducing sweating because of the epidermal impermeability of the macromolecule.

SC-injection of myricetin reduced sweating comparable to the effect of glycopyrrolate and BoNT/A. Sweating area was reduced to 13%, showing almost 2/3 sweat reduction. This dramatic reduction was also observed in the left footpads due to systemic effects (20% sweating area). However, skin-application of myricetin did not show such an antiperspirant activity on both footpads. It is thus likely that delivery of myricetin to sudomotor nerves was inefficient and that myricetin was oxidized by various cytochrome P450 isozymes present in the skin^31^. M-LNP showed 18% sweating area after SC-injection, which is equivalent to ~ 1/2 reduction of sweating. Systemic effect was also observed after SC-injection similarly to free myricetin, pilocarpine, and glycopyrrolate. When M-LNP was applied on the right footpads, sweating area was reduced to 18%, suggesting that LNP was effective in delivering myricetin to sudomotor nerves. M-LNP did not affect sweating on the left footpads suggesting its topical activity. In conclusion, among all the samples applied on the hind footpad skin of mice, only M-LNP reduced topical sweating.

### Cellular uptake of myricetin-loaded lipid nanoparticles

**Figure 3:**
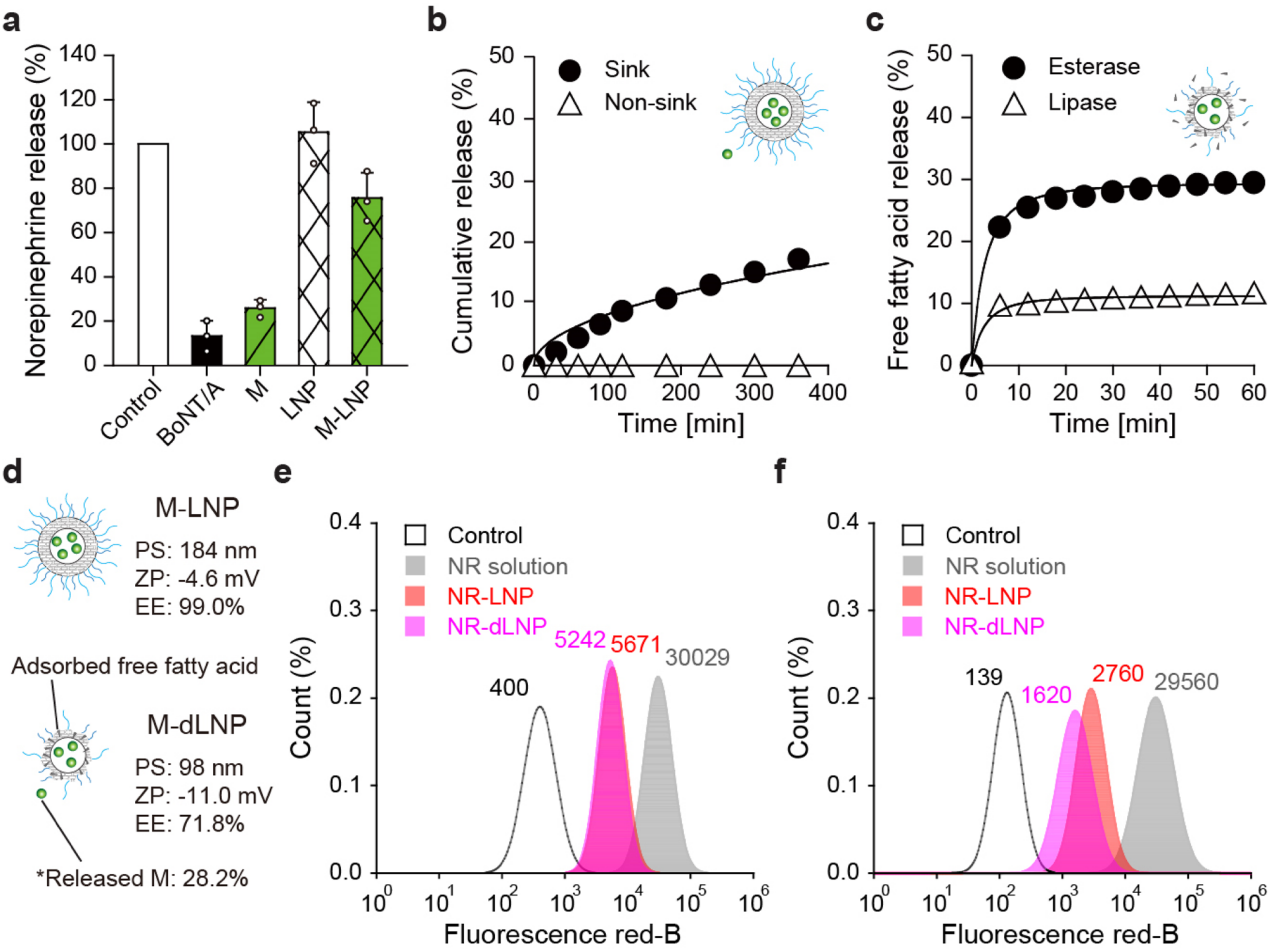
Cell uptake of M-LNP. (**a**) The % amount of norepinephrine released from differentiated PC12 cells after treatments with BoNT/A (50 pM), myricetin (M; 20 μM), blank-LNP (LNP), and M-LNP (20 μM) with respect to that from non-treated cells. Data are expressed as the mean ± s.d. (**b**) Cumulative amount of myricetin released from the M-LNP matrix in the sink (10 vol% ethanol in PBS at pH 5.0 and 37 °C) and non-sink conditions (PBS at pH 5.0 and 37 °C). (**c**) Quantification of free fatty acids released from the M-LNP matrix lipolyzed by esterase and lipase at pH 7.0 and 37 °C. (**d**) Physicochemical characteristics of M-LNP and M-LNP deformed by lipolysis (M-dLNP) (*PS* particle size, *ZP* ζ-potential, *EE* efficient efficiency). Flow cytometry of (**e**) the adult human dermal fibroblasts (HDFa) and (**f**) PC12 cells incubated with the culture media containing NR, NR-LNP, and NR-dLNP. Graphs were obtained from histograms in Supplementary Fig. S4d using fitting regression procedures.

The mechanism of inhibition of neuroexocytosis by M-LNP was investigated by measuring norepinephrine release from neuronal differentiated PC12 cells. Treatment with 50 pM BoNT/A inhibited as much as ~ 87% norepinephrine release consistently with our previous result (Fig. 3a)^18^. Treatment with 20 μM myricetin curtailed ~ 74% of norepinephrine release, which was comparable to the BoNT/A treatment. However, M-LNP reduced only ~ 24% of neurotransmitter release in vitro*,* contrary to that expected from the in vivo effect. This result raised the possibility that myricetin in M-LNP was restricted from cellular uptake. Besides, blank-LNP did not affect norepinephrine release, suggesting that the in vivo sweating-inhibitory activity of M-LNP did not resulted from the lipid matrix itself.

The lipid matrix of M-LNP was so stable in aqueous solution that the incorporated myricetin scarcely leaked out during storage for 6 months. Accordingly, only ~ 17% of myricetin in the M-LNP was discharged from the matrix after 6 h even in the sink condition that induced accelerated leaching of myricetin from M-LNP (Fig. 3b). Because several nonspecific esterases and lipases involved in skin triacylglycerol metabolisms are abundant in the skin, especially in the epidermis and skin appendages including sweat glands^32^. Breakdown of M-LNP was investigated in the presence of lipase or esterase (Fig. 3c). Esterase and lipase released ~ 29 and ~ 11% of FFAs from triacylglycerols of M-LNP, respectively, within 5 min of treatment, indicating rapid lipolysis of M-LNP in presence of lipolytic enzymes. As a result, M-LNP was deformed to show PS, ZP, and EE values of 98 nm, − 11 mV, and 71.8%, respectively, suggesting that some FFAs were adsorbed on the surface of deformed M-LNP (M-dLNP). Though myricetin scarcely leaked out from M-LNP, as much as 28.2% of myricetin was released after lipolysis in aqueous solution (Fig. 3d). In conclusion, the rate of myricetin discharge from M-LNP was greatly accelerated in the presence of esterase and lipase.

A lipophilic fluorescent dye, NR, was employed to simulate myricetin uptake into adult human dermal fibroblast (HDFa) and differentiated PC12 cells. After the cells were treated with NR solution, NR-loaded LNP (NR-LNP), and NR-dLNP (which was pretreated with a lipase), confocal microscopic images were taken. Fluorescence signals for NR were detected in the entire inner spaces of cells regardless of the incorporated forms and cell types, suggesting simple diffusional uptake into cells (Supplementary Fig. S2a,b, S3). Then, HDFa and PC12 cells were subjected to flow cytometry analysis to quantify the efficiency of NR entry into cells depending on the formulation (Fig. 3e,f). The fluorescence intensity of NR was observed in the order of NR solution, NR-LNP, and NR-dLNP for both cells. The fluorescence intensity of cells treated with NR solution were almost 5 times of those treated with NR-LNP or NR-dLNP, suggesting better cell uptake of free NR than of NR in LNPs. NR-dLNP showed lower fluorescence intensity possibly because the more negatively charged particles (− 11 mV) interfered with direct cellular uptake^33^. Therefore, it is likely that cellular uptake of myricetin in the form of intact M-LNP is just marginal, but can be rapidly accelerated by lipolytic deformation of LNP. In conclusion, the presence of esterase or lipase in the middle of transport routes from the stratum corneum to sudomotor neurons can promote the uptake of myricetin into skin cells including fibroblast and nerve cells.

### Localized sweating-inhibitory mechanism of myricetin-loaded lipid nanoparticles

Acetylcholine is released from postganglionic sympathetic nerve axons to sweat-secretory coils and activates myoepithelial and secretory cells in the coils resulting in perspiration^34^. Eccrine gland ducts directly open into the skin surface via sweat pores whereas apocrine gland ducts open into hair follicles. The coiled secretory tubules of both glands are located in the dermis. Secretory tubules are wrapped by myoepithelial cells joined with axons of sudomotor neurons^35^. Thus, myricetin can be delivered to neuronal axons either by (1) enabling it to penetrate all the way through the stratum corneum, epidermis, and dermis or (2) by transporting it along the hair follicle or sweat gland lumen, where it can be absorbed by neuronal axons.

**Figure 4:**
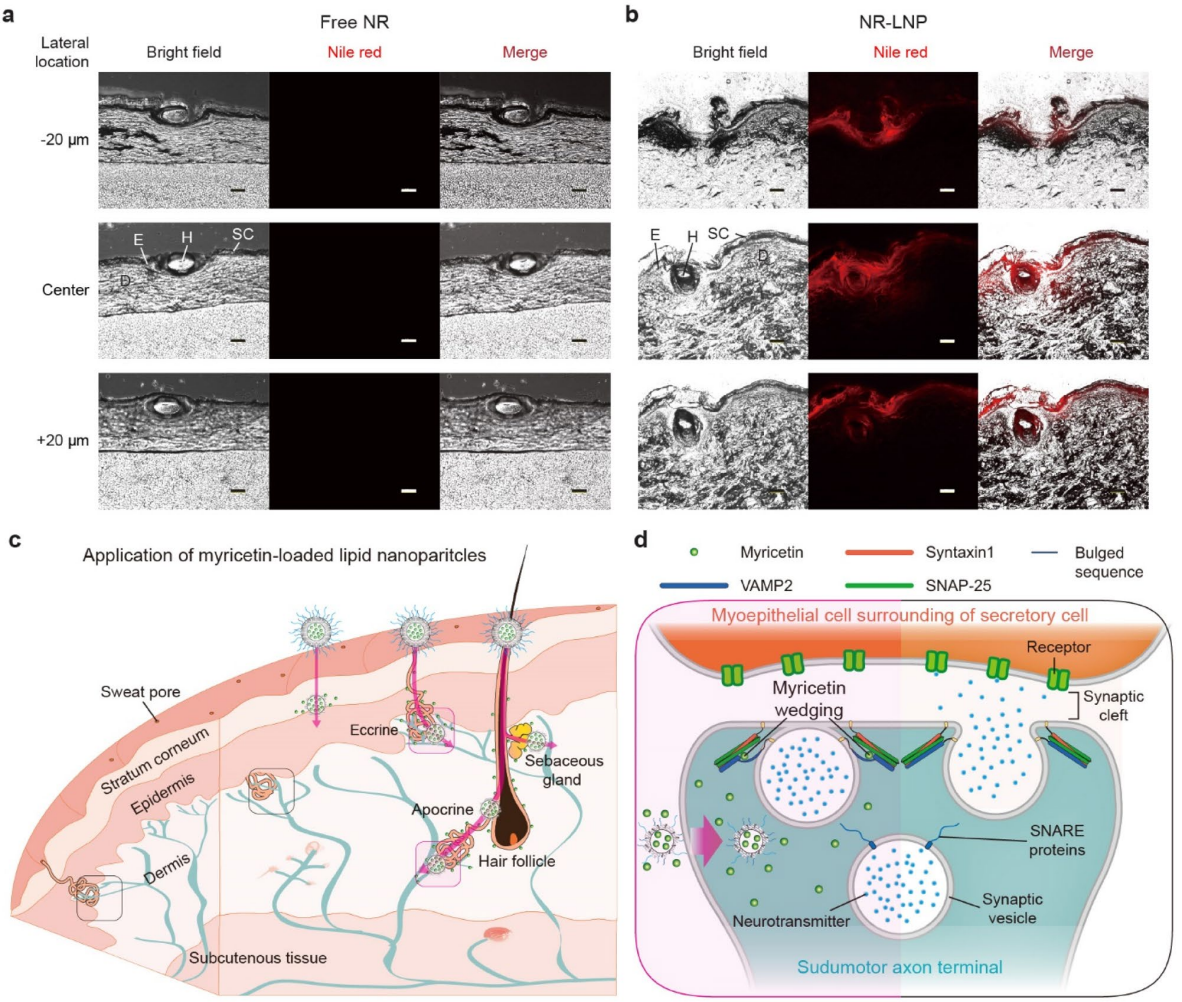
Transappendageal delivery of M-LNP. Fluorescence microscopic images of porcine skin vertically cryosectioned after incubation with (**a**) NR solution (5 vol% DMSO in PBS) or (**b**) NR-LNP (scale bars, 100 μm; *SC* stratum corneum, *E* epidermis, *D* dermis, *H* hair follicle). Thickness between neighboring cryosections was 20 μm. Schematic representations of (**c**) transdermal delivery (transappendageal and transepidermal routes) and (**d**) sweating-inhibitory activity of M-LNPs deformed by non-specific esterase present in the skin applied with M-LNPs.

The transport of myricetin within skin was simulated with NR applied on pigskin. After application of free NR and NR-LNP on pigskin, the distribution of NR was viewed with a fluorescence microscope after swiping out the NR remaining on the surface of pigskin. When free NR was applied to pigskin, NR was not detected in any of the pigskin slices which were 20 μm away from each other (Fig. 4a). In contrast, bright NR was observed after NR-LNP application on pigskin. NR was detected in the stratum corneum, epidermis, and even in the dermis of skin (Fig. 4b, Supplementary Fig. S5). Furthermore, a strong NR signal was detected in the area around hair follicles after NR-LNP application suggesting that NR-LNPs travelled along the gland ducts. This result suggests the feasibility of M-LNP for delivery through both transepidermal and transappendageal pathways, consistent with conventional lipid-carrier systems.

Because corneocyte-layers are dense and the intercellular lipid-lamella in the stratum corneum are narrow^36^. It is unlikely that M-LNP with a diameter of ~ 200 nm penetrated several layers to the dermis. It is also not likely that myricetin passively diffused to the dermis after discharging from M-LNP because the LNP was very stable in solution. Although LNP itself can help skin permeation of myricetin because intact or deformed M-LNP can attach to the skin surface and increase skin hydration, which gradually loosens the intercellular structures of stratum corneum^36^, intact LNPs are not sufficiently deformable to penetrate via the intercellular spaces of the stratum corneum unlike liposomes, due to their solid-state^37^. As shown above, LNP or dLNP structures were not well-absorbed by the cells. Thus, we propose a working model for the delivery of myricetin to sudomotor nerves and subsequent anti-perspiration activity (Fig. 4c,d). First, some of the M-LNPs applied to skin are transported through skin-appendages (hair follicles, sweat glands, and sebaceous glands) whereas the other M-LNPs remain on the skin surface. Second, myricetin is released from M-LNPs by lipases and esterases. Several nonspecific esterases and lipases are abundant in skin-appendages^32^. Human skin esterase is known to have high enzymatic activity^38^. Third, myricetin discharged from M-LNPs in the bottom of skin-appendages can reach sudomotor nerves whose neuroexocytosis is regulated by inhibition of SNARE complex formation (Fig. 4d). In conclusion, the LNP is an efficient delivery vehicle enabling modulation of sudomotor nerve excitation by combining stability in solution and drug release by the lipases and esterases abundant in skin-appendages.

### Biocompatibility of myricetin-loaded lipid nanoparticles

**Figure 5:**
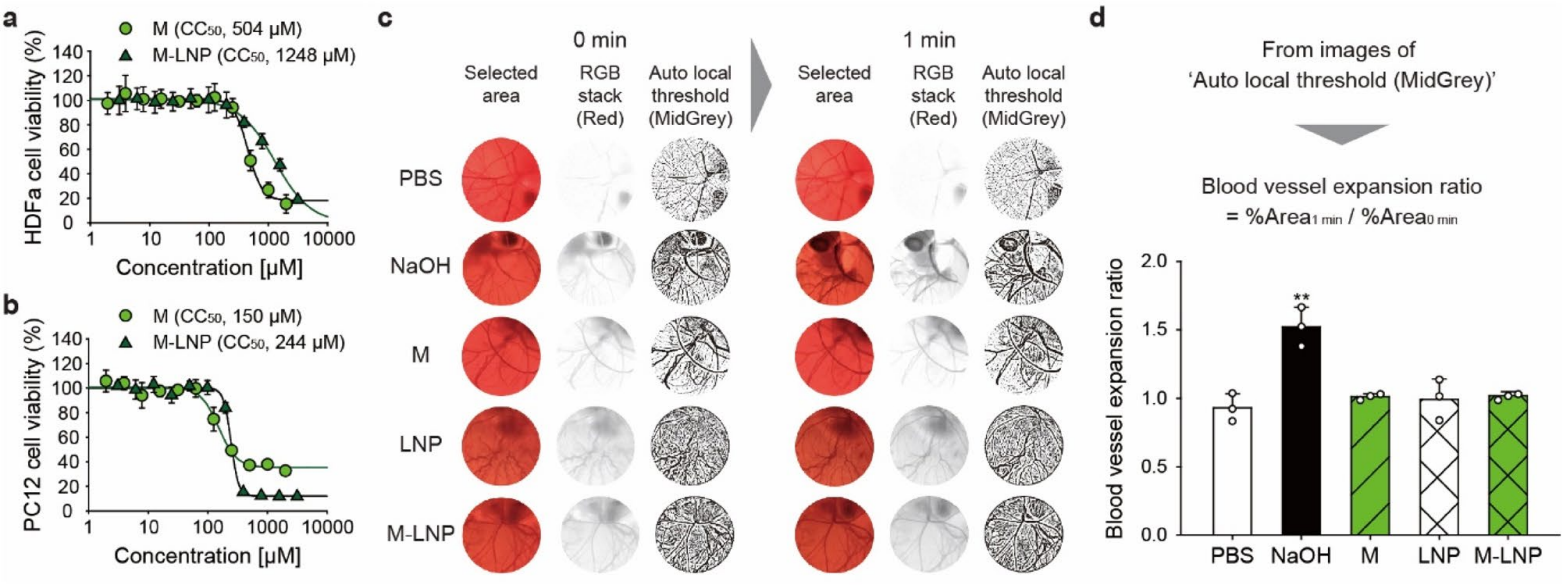
Toxicity of M-LNP. Viabilities (%) of (**a**) adult human dermal fibroblasts (HDFa) and (**b**) PC12 cells incubated with myricetin (M) and M-LNP were measured by the AlamarBlue cell viability assay. Fifty percent cytotoxic concentrations (CC_50_) were determined using a sigmoid fitting model. All data are expressed as the mean ± s.d. (**c**) Determination of blood vessel area from hen’s egg chorioallantoic membrane images obtained before and 1 min-after the 2 mL-sample treatments, using image processing procedures in ImageJ. Samples: 5 vol% dimethyl sulfoxide in phosphate buffered saline (PBS), 1 M NaOH aqueous solution (NaOH), M solution (M: 0.1 mg mL^−1^ in 5 vol% DMSO in PBS), and Blank-LNP (LNP) and M-LNP diluted tenfold with PBS (M: 0.1 mg mL^−1^). (**d**) Blood vessel expansion ratios at 1 min after the sample treatments. Percentages of black pixel fraction (%Area) were determined from images of ‘Auto local threshold (MidGrey)’ at 0 and 1 min. Data are expressed as the mean ± s.d. (Student’s *t*-test; **p < 0.01).

To assess the biocompatibility of M-LNPs, their cytotoxicity was measured using differentiated PC12 and HDFa cells. The 50%-cytotoxic concentrations (CC_50_) of myricetin to PC12 and HDFa cells were 150 and 504 μM, respectively, whereas those of M-LNPs were 244 and 1,248 μM, respectively (Fig. 5a,b). M-LNPs showed 1.5–2.5 times higher CC_50_ values than myricetin, indicating that encapsulation could make myricetin safer than the free form. Indeed, lipid matrices of the LNP were prepared using TS, TC, BR, and PL, which are known to be biocompatible. Moreover, the LNP system is known to have low toxicity^39^ and even reduces the cytotoxicity of a drug^40^ whereas some nanoparticles may show cytotoxicity^41^. The hen’s egg chorioallantoic membrane test (HET-CAM) showed that blood vessels were neither expanded nor shrunk after treatments with myricetin, blank-LNP, and M-LNP (Fig. 5c,d). Hemorrhage, blood-coagulation, and hyperaemia were not observed (Supplementary Table S2), implying that there is no ocular irritation in accordance with a previous study on epigallocatechin gallate-loaded LNPs^42^. These results suggest that M-LNPs are an effective and safe nanoparticle that can be applied on skin for treating topical hyperhidrosis.

## Conclusions

Inspired by the successful treatment of hyperhidrosis with the SNARE-specific protease BoNT/A, M-LNP was fabricated to achieve a localized antiperspirant effect using the SNARE complex-inhibitory flavonoid, myricetin. M-LNP was stable in solution, but released myricetin in the sweat gland after lipolysis by lipases/esterases. This study revealed that myricetin loaded in the matrices of the LNPs was transported from the skin surface to the sudomotor nerves that stimulate sweat glands in the following sequence: (1) transappendageal transportation of the M-LNPs, (2) lipolysis of the matrix, and release of myricetin, (3) diffusion of myricetin to the nerves, and (4) inhibition of the formation of the SNARE complex. The results of the cytotoxicity and HET-CAMs indicated that M-LNPs are safe for use in the clinic. Taken together, this M-LNP strategy would serve as a promising approach for the sudomotor nerve-targeted delivery of myricetin via the transappendageal pathway, exerting topical sweat inhibitory effects unlike the conventional anticholinergic agents. The M-LNP strategy shows great potential as an effective, safe, and patient compliance-enhancing treatment for focal hyperhidrosis.

We expect that myricetin and M-LNP demonstrated in this study provide useful tool for studying the underlying mechanism of antiperspirant activity of myricetin but also fundamentals of neurotransmission. Paul et al. have shown that laccase not only degrades free myricetin but also removes SNARE-bound myricetin^17^. Thus, it is highly possible that laccase delivered to skin appendages help to demonstrate that myricetin encapsulated in LNPs acts directly on the sudomotor nerve terminals. It is possible that degradation of myricetin by laccase outside nerve cells can mitigate M-LNP effect upon myricetin discharge from LNP. Unfortunately, however, we failed to test this idea because of strong cytotoxicity of laccase through unknown reason. Just submicromolar concentration of laccase resulted in death of PC12 cells (data not shown). In addition, delivery of laccase to the bottom of skin appendages is not trivial because (1) laccases (molecular weight of ~ 140 kDa) are too big to penetrate dense dead cell blocks and narrow intercellular spaces in the stratum corneum covering the epidermis. Stratum corneum blocks most macromolecules and even small chemicals diffusing into underneath the dermis through it^43–45^, (2) there are strong proteases in the skin appendages, and (3) laccase cannot be encapsulated in the LNP used in this study. Hydrophobic core of LNP does not contain water-soluble laccase. Furthermore, heating during the LNP formation procedure denatures laccase. Thus, development of a transdermal delivery system for laccase will help to test stop-and-go of SNARE complex formation in the sudomotor nerves.

It is expected that the myricetin results in hemifusion between synaptic vesicle and presynaptic cell membrane if the effect of myricetin on liposome-liposome fusion is also valid inside nerve cells^17,18^ However, hemifusion inside nerve cells is still controversial. A conical electron tomography has shown that vesicles docked to the active zone are hemifused^46^. Granules in the sea urchin egg has been shown to stably hemifuse with the plasma membrane using fluorescence recovery after photobleaching^47^. However, the other cryoelectron tomography study has proposed that docked synaptic vesicles did not make membrane-to-membrane contact with the active zone^48^. Myricetin and M-LNP seems to provide a great opportunity to answer the controversial question whether the hemifused state may correspond to the primed vesicle pool. Myricetin will stabilize hemifusion if the structure is valid in the cell. M-LMP is likely to help to observe hemifusion in the delivered tissue not restricted to cell model. Mouse footpad treated with M-LNP will be a good tissue model for studying hemifusion inside cell. Even though these works are beyond the scope of current study, elegant electron microscopy studies may take advantages of myricetin and M-LNP to show the ambiguous membrane structure.

## Methods

### Chemicals

Myricetin, TS, TC, BR, PL, NR, hepatic esterase from porcine (≥ 15 U mg^−1^ solid), and pancreatic lipase from porcine (Type II, 100–500 U mg^−1^ protein) were purchased from Sigma Aldrich Co. (St. Louis, MO, USA). Dulbecco’s modified Eagle medium (DMEM), fetal bovine serum (FBS), and trypsin–EDTA were purchased from GE Healthcare (Chicago, Illinois, USA). Pilocarpine HCl and glycopyrrolate bromide were obtained from Tokyo Chemical Industry (Tokyo, Japan). Meditoxin, alfaxalone (Alfaxan), and xylazine HCl (Rompun) were purchased from Medy-Tox Inc. (Ochang, Korea), Careside (Gyeonggi-do, Korea), and Bayer Korea (Seoul, Korea), respectively. Antibiotic–antimycotic (10 ×), Medium 106, and low serum growth supplement kit were purchased from Gibco/Invitrogen (Grand Island, NY, USA), and AlamarBlue cell viability reagent was obtained from Thermo Scientific (Waltham, Massachusetts, USA). All other chemicals were of analytical and reagent grade.

### Preparation of lipid nanoparticles

Myricetin-loaded LNPs were prepared using an oil-in-water emulsion technique with a high-speed blender and sonication probe as reported previously^28^, with slight modifications. First, the lipid (5 wt%) and aqueous (95 wt%) phases were heated to 85 °C and mixed using a high-speed blender (Ultra-Turrax T25D,Ika Werke GmbH & Co., Staufen, Germany) at 8,000 rpm for 1 min and then at 11,000 rpm for 1 min. The lipid phase of M-LNPs was prepared by blending the TS (68.6 wt% of the lipid phase), TC (29.4 wt% of the lipid phase), and myricetin (2 wt% of the lipid phase) dissolved in ethanol (25 mg mL^−1^) and subsequently evaporating the ethanol with stirring for 30 min (85 °C). The aqueous phase was fabricated by dissolving BR (7.5 wt% of the entire M-LNPs) and PL (7.5 wt% of the entire M-LNPs) in double-deionized water (DDW) with stirring for 12 h. After preparing the coarse oil-in-water emulsion, droplet size was further reduced by sonication (VCX 750; Sonics & Materials Inc., Newtown, CT, USA) for 4 min at 60% amplitude, a duty cycle of 1 s, and 95 °C. Next, post-sonication for 6 min was applied to the emulsions during cooling to 45 °C in a jacketed beaker, and the resulting M-LNP samples were immediately stored at 4 °C. NR-LNPs were prepared in the same manner as M-LNPs, except that they included NR at the same composition instead of myricetin.

### Physicochemical characterization of the lipid nanoparticles

To assess physicochemical characteristics of the LNPs, experimental and measuring methods in this section were applied using the methods previously introduced^28^. LNPs diluted tenfold with DDW were passed through a 1-μm pore-size glass microfiber filter (GF/B; Whatman Ltd., Loughborough, UK). The aggregated LNPs (> 1 μm) remaining on the filter were weighed after drying in an oven at 60 °C. The difference in filter weight before and after this procedure, which was the weight of the aggregated LNPs, was recorded, and the yield value was then calculated as the percentage of the difference over the sum of the weight of the LNPs. For measuring the PS (z average) and ZP for LNPs passed through the filters, a Zetasizer (Nano ZS90; Malvern Instruments Ltd., Worcestershire, UK) was used and operated at a 90° angle with a helium–neon laser (λ = 633 nm). The ZP measurement was based on the Smoluchowski equation at 25 °C with an electric field strength of 20 V cm^−1^.

Assuming that all particles are spherical, Γ_s_ was calculated as ${\Gamma }_{\mathrm{s}}={C}_{\mathrm{a}}D/6\Phi$, where ${C}_{\mathrm{a}}$ is the concentration of the surfactant adsorbed to the surface of LNPs, $D$ is the PS, and Φ is the lipid phase volume fraction (i.e., 0.05)^28,49^. The method for determining ${C}_{\mathrm{a}}$ values was described previously^50^. Briefly, surfactants not covering the lipid particles were collected using a centrifugal ultrafiltration with Sartorius Vivaspin 500 polyethersulfone micro-concentrators (molecular weight cut-off, 10 kDa; Göttingen, Germany) and were quantified using a spectrophotometer (Pharmaspec UV-1700; Shimadzu Corp., Kyoto, Japan) and ammonium cobaltothiocyanate solution. The ${C}_{\mathrm{a}}$ values for the LNP system were calculated by subtracting the non-covering surfactant concentration from the entire concentration of surfactants used.

A 1-mL quantity of M-LNPs was carefully added on a layer of 30 wt% sucrose aqueous solution in a 2 mL micro-tube. Without any layer mixing, the micro-tube was centrifuged at 25,000 relative centrifugal force (RCF) for 30 min (Eppendorf 5427R; Eppendorf AG, Hamburg, Germany). After centrifugation, the upper layer for M-LNPs was carefully removed, and 0.25 mL of the sucrose-solution layer containing the precipitated pellet was collected and mixed with 0.75 mL methanol under sonication treatment. After centrifugation at 25,000 RCF for 10 min, the supernatant was used as a sample for myricetin quantification with a spectrophotometer (standard curve, 0.5‒16 μg mL^−1^, *R*^2^ = 1.0000; *λ* = 375 nm). EE for the M-LNPs was determined using the equation:

1 $$\mathrm{EE }(\mathrm{\%})=100\times ({\mathrm{W}}_{\mathrm{t}}-{\mathrm{W}}_{\mathrm{s}})/{\mathrm{W}}_{\mathrm{t}}$$

where ${\mathrm{W}}_{\mathrm{t}}$ is the total myricetin molecule weight in the entire system, and ${\mathrm{W}}_{\mathrm{s}}$ is the myricetin molecule weight in the supernatant.

### Transmission electron microscopy

Morphologies of LNPs were observed using energy-filtering TEM (LIBRA 120; Carl Zeiss, Oberkochen, Germany). For the observation, a drop of LNPs diluted 20-fold with DDW was placed on a film-coated copper grid and was negatively stained with a 1% w/v aqueous solution of phosphotungstic acid for 30 s. Next, the excess solution was wiped off and the grid was dried for a day at 25 °C before observation.

### Deferential scanning calorimetry

Polymorphisms of the bulk lipid phase, the aqueous phase, Blank-LNPs, and M-LNPs were determined using a DSC (Diamond DSC; PerkinElmer, Waltham, MA, USA). Each sample (20 ± 5 mg) placed in a hermetic aluminum pan, which was sealed and equilibrated at 25 °C overnight prior to measurement. An empty pan was used as a reference. The DSC scan started at 25 °C, increased by 5 °C min^−1^ to 95 °C and then decreased by 5 °C min^−1^ to 10 °C.

### Sweat assay

Six week-old male ICR (Institute of Cancer Research) mice weighing 25–35 g were purchased from Koatec Co. (Pyeongtaek, Korea) and tested in accordance with the approval and guidelines of the Institutional Animal Care and Use Committee of Sungkyunkwan University (SKKUIACUC2018-04-11-2). All mice associated with this study were housed in a facility at Sungkyunkwan University, and all experimental protocols were approved by animal care and use committee of Sungkyunkwan University. Seventy five mice were randomly divided into 15 groups (5 mice per group). Then, 14 groups except a sample non-treated group, were divided into two subgroups according to the treatment methods as SC injection and skin-application, and the samples used for each treatment method were as follows. PBS (pH 7.4), 0.8 mL kg^−1^; negative control (−), 2.5 mg kg^−1^ of pilocarpine HCl (3.125 mg mL^−1^ PBS); positive controls ( +), 0.25 mg kg^−1^ of glycopyrrolate bromide (312.5 μg mL^−1^ PBS) and 0.8 U kg^−1^ of Meditoxin (1 U mL^−1^ PBS); myricetin, 0.8 mg kg^−1^ of myricetin [1 mg mL^−1^ 5% dimethyl sulfoxide (DMSO) in PBS]; LNPs, 0.8 mL kg^−1^; and M-LNP, 0.8 mL kg^−1^ (based on the amount of myricetin as 1 mg mL^−1^).

The assay methods used in this study were slightly modified from those described previously^51^. Mice were anesthetized for > 1 h, using intraperitoneal injection of the solution mixed with 80 mg kg^−1^ Alfaxan and 10 mg kg^−1^ Rompun. Next, samples were either applied onto right hind footpads of anesthetized mice or SC-injected into the right hind footpads without any damage. Mice treated with the methods of application and SC-injection were maintained without interruption for 30 and 10 min, respectively, to allow a sufficient time for absorption and action of the agents. After wiping off the samples on footpads, both left and right hind footpads were painted with 3.5% w/v iodine solution (in ethanol), allowed to dry for 1 min, and coated with 10% w/v starch solution (in castor oil). Immediately after starch solution coating, footpad photographs were taken with a 12-megapixel digital camera under fixed conditions of distance, magnification, and exposure.

### Cumulated release of myricetin from lipid nanoparticles

Cumulative release pattern of myricetin from the lipid matrix of M-LNPs was examined using dialysis bags with a 12-kDa molecular weight cut-off. The bag was immersed in DDW for 12 h prior to use. The bag was filled with 3 mL of M-LNP, tightly sealed, and suspended in 45 mL of 10 vol% ethanol in PBS at pH 5.0 (to prevent the myricetin oxidation) with stirring at 100 rpm and 37 °C. At predetermined time intervals, 1 mL aliquots were replaced with 1 mL of fresh PBS (10 vol% ethanol). The absorbance of the aliquot was recorded with a spectrophotometer. Concentrations of myricetin in the aliquots were calculated using a standard curve ranging from 2.5 to 40 μg mL^−1^ (λ = 375 nm, *R*^2^ = 0.9999). The obtained cumulative release profile was fitted using the Higuchi equation ($Q={K}_{\mathrm{H}}{t}^{1/2}$; $Q$, cumulative percentage of myricetin release; ${K}_{\mathrm{H}}$, Higuchi constant; $t$, time in minutes)^52^.

### Monitoring the lipolysis of lipid nanoparticles

The lipolysis of LNPs in skin was monitored in the in vitro simulated fluid using the previously reported titration method, with slightly modifications^50^. The simulated fluid was prepared by dissolving sodium chloride, calcium chloride, and either hepatic esterase or pancreatic lipase in a 10 mM sodium phosphate buffer (pH 7) at concentrations of 48.8, 11.1, and 6 mg mL^−1^, respectively, and was kept at 37 °C prior to the experiments and was adjusted to a pH of 7 with 2.5 M NaOH aqueous solution as necessary. Before titration, the LNPs were diluted tenfold with 10 mM sodium phosphate buffer (pH 7), which were maintained at 37 °C and adjusted to pH 7 with 2.5 M NaOH aqueous solution if necessary. Finally, based on the concepts that hepatic esterase and pancreatic lipase hydrolyze a triacylglycerol to a diacylglycerol/a FFA and a monoacylglycerol/two FFAs, respectively, the lipolysis of diluted LNPs (20 mL) was monitored by measuring the released FFA after the addition of simulated fluid (5 mL). The released amount of FFAs was quantified by adding 50 mM NaOH aqueous solution to the reaction vessel (37 °C to maintain the pH of the solution at 7 using an automatic titration unit (842 Titrando; Metrohm AG, Herisau, Switzerland. The measured amount was converted into a $\mathrm{\%FFA}$ value with the following equation:

2 $$\mathrm{\%FFA}=100\times ({V}_{\mathrm{NaOH}}\times {m}_{\mathrm{NaOH}}\times {M}_{\mathrm{Lipid}})/({w}_{\mathrm{Lipid}}\times {N}_{\mathrm{FFA}})$$

where ${V}_{\mathrm{NaOH}}$ is the volume (mL) of the NaOH solution required to neutralize the released FFAs, ${m}_{\mathrm{NaOH}}$ is the molarity (M) of the NaOH solution, ${M}_{\mathrm{Lipid}}$ is the molecular weight (0.7 × 891.48 + 0.3 × 470.69 = 765.24 g mol^−1^) of the lipid mixture (TS and TC), ${w}_{\mathrm{Lipid}}$ is the total weight (0.1 g) of the lipid mixture initially present in the reaction vessel, and ${N}_{\mathrm{FFA}}$ is the expected number of FFA produced from a triacylglycrol (hepatic esterase, 1; pancreatic lipase, 2). Blank experiments were also conducted with heat-inactivated enzymes (95 °C, 15 min) to eliminate any pH decrease due to other factors. Calculated values for $\mathrm{\%FFA}$ were fitted using regression curves as follows^53^:

3 $$\mathrm{\%FFA}={\%\mathrm{FFA}}_{\mathrm{max}}\left\{1-{\left(1+3k{M}_{\mathrm{Lipid}}t/2D{\rho }_{\mathrm{Lipid}}\right)}^{-2}\right\}$$

where ${\%\mathrm{FFA}}_{\mathrm{max}}$ is the $\mathrm{\%FFA}$ that has been hydrolyzed by the end of the lipolysis process, $k$ is the rate constant (μmol s^−1^ m^−2^), $t$ is the time (s), and *ρ*_Lipid_ is the density of the lipid mixture (0.7 × 0.86 + 0.3 × 0.95 = 0.89 g mL^−1^).

The reaction was terminated by adding 60.25 μL of 4-bromophenylboronic acid solution (0.5 μM) at the end of lipolysis. The soluble and insoluble fractions of myricetin in the lipolyzed solutions were separated for 10 min at 15,000 RCF (25 °C) using a centrifuge (Supra 22K; Hanil Science Industrial Co., Seoul, Korea). Twelve milliliters of the supernatant (soluble fraction) were mixed with 20 mL methanol and the pellet (insoluble fraction) was blended with 20 mL methanol and 20 mL sodium phosphate buffer (10 mM, pH 7). The mixtures containing the supernatant and pellet were sonicated for 1 min to solubilize myricetin into solvents and were centrifuged again to remove the residual substances (proteins, enzymes, and other residues). Finally, the amount of myricetin in the second supernatants was quantified using a spectrophotometer. Then, the PS and ZP of the lipolyzed LNPs present in the second supernatant for the soluble fraction were measured using the Zetasizer.

### Cell culture

PC12 cells obtained from Korean Cell Line Bank (KCLB, Seoul, Korea), were cultured in media (89 vol% DMEM, 10 vol% FBS, and 1 vol% antibiotic–antimycotic solution). HDFa (GIBCO/Invitrogen, Carlsbad, CA, USA) were cultured in supplemented medium 106 with low serum growth supplement kits using the vendor’s protocol, and were harvested using trypsin–EDTA. All cells (1–5 passages) cultivated in this study were grown under incubation (37 °C, 5% CO_2_) and the culture media were replaced every other day.

### Quantification of norepinephrine release

To quantify the release of norepinephrine from PC12 cells, compositions and treatments of the solutions used in this assay was slightly modified from the method previously reported by our group^18,54^. Harvested PC12 cells were seeded onto 24-well plates coated with poly-d-lysine at a density of 2 × 10^5^ cells per well and were grown in the culture media for 5 days (37 °C, 5% CO_2_). Then, the nerve growth factor (50 ng mL^−1^; NGF mouse protein 7S subunit; GIBCO/Invitrogen, Carlsbad, CA, USA) was added and incubated again for 7 days. After incubation, 1 mL of high-K^+^ medium (115 mM NaCl, 50 mM KCl, 1.2 mM KH_2_PO_4_, 2.5 mM CaCl_2_, 1.2 mM MgSO_4_, 11 mM glucose, and 15 mM Hepes-Tris; pH 7.4) was added to the well and washed with the low-K^+^ medium (140 mM NaCl, 4.7 mM KCl, 1.2 mM KH_2_PO_4_, 2.5 mM CaCl_2_, 1.2 mM MgSO_4_, 11 mM glucose, and 15 mM Hepes-Tris; pH 7.4) after 15 min. After washing, 1 mL of 1 vol% DMSO in the low-K^+^ medium, containing the inhibitor sample, was added to the well and incubated for 4 h (37 °C, 5% CO_2_) (final concentrations of Meditoxin, myricetin solution, and myricetin of M-LNP: 50 pM, 20 μM, and 20 μM, respectively). Next, the cells were washed to remove the extracellular compounds with the low-K^+^ media and treated again with the high-K^+^ solution to depolarize the cells and stimulate neurotransmitter release. After 15 min in the high-K^+^ medium treatment, neurotransmitters released from the cells into the medium were quantified using the norepinephrine enzyme-linked immunosorbent assay kits (IBL International, Hamburg, Germany) and a microplate reader (*λ* = 405 nm; Synergy H1; BioTek Instruments Inc., VT, USA). Consequently, the amount of released norepinephrine was determined as the subtraction of the basal level signal from the signal of sample-treated cells.

### Flow cytometry

Harvested PC12 or HDFa cells (2.0 × 10^5^ cells) were incubated for 4 h (37 °C, 5% CO_2_) with 2 mL of culture medium containing NR (0.01 mg mL^−1^), which was dissolved in DMSO or incorporated in intact or lipolyzed LNPs (all the samples tested were pre-passed through 1 μm-syringe filters). The incubated cells were centrifuged (200 RCF, 3 min) and washed with PBS; this was repeated four times. Subsequently, the cells (1.0 × 10^5^ cells mL^−1^) suspended in PBS were loaded in a flow cytometer (guava easyCyte System; Millipore, MA, USA) to acquire fluorescence signals (red-B) of the NR within the cells. The obtained data were analyzed using FlowJo software (Version 10; FlowJo LLC, OR, USA). Based on the forward scatter-versus-side scatter plots, the signals from only the cells (> 1,400) were collected and histograms and medians thereof were obtained on the software. The histograms were modeled with the normal log peak model in SigmaPlot 10.0 Windows version (IBM Co., Armonk, NY, USA).

### Confocal laser fluorescence microscopy

Harvested PC12 cells (2.0 × 10^4^ cells well^−1^) were seeded onto 12-well plates containing poly-D-lysine-coated coverslips, incubated in PC12 culture medium to adhere to the coverslips (5 day, 37 °C, 5% CO_2_), treated with nerve growth factor (10 ng mL^−1^), and incubated again to differentiate for a week. Harvested HDFa cells (2.0 × 10^4^ cells well^−1^) were seeded on 12-well plates containing coverslips and incubated in HDFa culture medium (7 days, 37 °C, 5% CO_2_). The cells in wells were further incubated for 4 h with culture medium containing NR (0.01 mg mL^−1^), which was dissolved in DMSO or incorporated in intact or lipolyzed LNPs (all samples tested were pre-passed through a 1 μm-syringe filter. After incubation, the cells were washed with PBS, fixed for 15 min with 3.7 wt% paraformaldehyde in PBS, and permeabilized for 5 min with 0.5 vol% Triton X-100 in PBS. Nuclei, filamentous actin, and glycolipids/glycoproteins in the cells were stained with Hoechst 33258 (Invitrogen), Oregon Green 514 phalloidin (Invitrogen), and WGA-AF647 (wheat germ agglutinin Alexa Fluor 647 conjugate; Invitrogen), respectively. After washing with PBS (× 3), the coverslips were mounted with ProLong Gold Antifade Mountant (P10144; Molecular Probes, Eugene, OR, USA). Images were acquired using a Leica TCS SP8 HyVolution confocal microscope with an × 63 objective (HC PL APO 40 × /1.10 W CORR CS2, FWD = 0.65 mm; Leica Microsystems, Wetzlar, Germany). All immunofluorescence images were acquired at 25 °C. Images presented together were processed identically.

### Epidermal permeation test

Excised miniature pig skin (Micropig Franz cell membrane; Medi Kinetics Co., Ltd., Gyeonggi-do, Korea) was mounted between the donor and receptor compartments of Franz diffusion cells (Permegear Inc., Riegelsville, PA) with the stratum corneum facing the donor compartment (diffusional surface area, 0.636 cm^2^). NR solution (1 mg mL^−1^ 5 vol% DMSO in PBS) and NR-LNP were added to the epidermal side in the donor compartment and the receptor compartment was filled with 10 vol% ethanol in PBS (pH 7.4) maintained at 37 °C. The epidermal permeation studies were conducted under un-occlusive conditions where water evaporation was allowed. After 24 h from the addition of each sample, the skin was displaced from the diffusion cell and dry-wiped, and the entire dosing area was punched out. After embedding in optimal cutting temperature compound (Sakura Finetek, Torrance, CA, USA) and freezing in liquid nitrogen, the center of collected skins was cryosectioned vertically (thickness, 20 μm) at − 28 °C and stored at − 80 °C. The skin sections were visualized with a fluorescence microscope (SMZ1000; Nikon Corporation, Tokyo, Japan).

### Cell viability determination

Cell viability was determined using the AlamarBlue cell viability assay protocol provided by the vendor. Briefly, harvested cells (1.0 × 10^4^ per well), of which viability was assessed using trypan blue, were seeded in the wells of a 96-well plate and incubated in culture medium (37 °C, 5% CO_2_) for attachment to the plate. After 5 days, the media were replaced with sample solutions appropriately diluted with culture media and incubated for 12 h (37 °C, 5% CO_2_). Next, after removing the culture medium, the cells were quickly rinsed with PBS and incubated again (37 °C, 5% CO_2_) with 100 μL of FBS-free culture media and 10 μL of AlamarBlue cell viability reagent. After 2 h of re-incubation, fluorescence was measured under the fluorescence mode of the Synergy H1 microplate reader (*λ*_ex_/*λ*_em_ = 560 nm/595 nm).

### Hen’s egg chorioallantoic membrane test

The HET-CAM is an assay well-correlated with ophthalmic irritancy in vivo^55^. For HET-CAMs, fertilized eggs were obtained from a local market in Korea and incubated for 10 days (37.5 °C and 45% humidity), with auto-rotation at 90° h^−1^. After incubation, the upper shell of the embryo-formed eggs was cut quickly in a circular shape with caution not to damage the inner membrane. Immediately after cutting, the inner membrane was carefully removed with forceps to avoid injury to the blood vessels, and test samples of 2 mL were dispensed onto the CAM. Here, right before sample treatment, the myricetin sample was prepared by mixing 1.9 mL of PBS and 100 μL of myricetin solution (2 mg mL^−1^ in DMSO), and Blank-LNP and M-LNP was diluted tenfold with PBS. PBS and 1 M NaOH solution were used as the positive and negative controls, respectively. Next, using a flashlight contacted with the bottom eggshell, CAM images were obtained after 1 min from sample addition. Based on the CAM images obtained, the severity of any hemorrhage, blood-coagulation, and hyperaemia was graded from 0 (no irritation) to 3 (strong irritation) using the scoring method developed previously^55^.

### Image processing

Images obtained from the sweat assay and the HET-CAM were further processed using ImageJ (available as freeware from https://rsb.info.nih.gov/ij/) to prevent arbitrary judgments. For the sweat assay, the images were loaded individually into ImageJ, cropped for size to include only the footpad area, and converted to greyscale by selecting the menu item ‘Image’ > ‘Type’ > ‘RGB stack’. The greyscale images on red stack were adjusted by selecting the menu item ‘Image’ > ‘Adjust’ > ‘Auto Local Threshold’ with the Phansalkar method. Finally, the area fraction (%Area) values of black pixels in the adjusted images were measured and recorded after pressing the ‘Ctrl + M’ key on a keyboard, which could represent the sweating area. For the HET-CAM, CAM images were loaded individually into ImageJ, cropped for size to exclude the shell, and converted to greyscale. The greyscale CAM images on red stack were adjusted with the MidGrey method. Finally, the %Area values of adjusted CAM images were measured and recorded. Ratios of the %Area after versus that before the sample treatments were defined as the blood vessel expansion ratios.

### Statistical analyses

All data represent an average of at least three independent experiments or measurements. The results were reported as averages and standard deviations of these measurements. The kinetic parameters and curves were estimated and fitted using the regression iteration procedures in SigmaPlot 10.0. Statistical analyses, Student’s *t*-tests, were conducted using SPSS Statistics version 23.0 software (IBM Co., Armonk, NY, USA).

## Supplementary information

Supplementary Information
