# Supplementary material for: Reduction of focal sweating by lipid nanoparticle-delivered myricetin
Source: Sci Rep. 2020 Aug 4;10:13132. doi: 10.1038/s41598-020-69985-x (PMC7403431; doi:10.1038/s41598-020-69985-x)
Supplement: Supplementary file 1 — Supplementary Information [file 41598_2020_69985_MOESM1_ESM.pdf]

Reduction of focal sweating by lipid nanoparticle-delivered myricetin

Choongjin Ban[1†], Joon-Bum Park[2†], Sora Cho[3], Hye Rin Kim[2], Yong Joon Kim[2], Young Jin Choi[6,7,8], Woo-Jae Chung[2,3,4,5]*, Dae-Hyuk Kweon[2,3,4,5]*

[1]Department of Environmental Horticulture, University of Seoul, 163 Seoulsiripdaero, Dongdaemun-gu, Seoul 02504, Republic of Korea

[2]Department of Integrative Biotechnology, [3]Interdisciplinary Program in BioCosmetics, [4]Institute of Biomolecule Control, and [5]Biologics Research Center, Sungkyunkwan University, 2066 Seoburo, Suwon, Gyeonggi 16419, Republic of Korea

[6]Department of Agricultural Biotechnology, [7]Center for Food and Bioconvergence, and [8]Research Institute of Agriculture and Life Sciences, Seoul National University, 1 Gwanakro, Gwanakgu, Seoul 08826, Republic of Korea

*Corresponding authors:

Woo-Jae Chung (Email: wjchung@skku.edu) and Dae-Hyuk Kweon (Email: dhkweon@skku.edu).

[†]These authors contributed equally to this work.

Table S1. Yield, entrapment efficiency (EE), particle size (PS), ζ-potential (ZP), and surface load (Γ_s) of the lipid nanoparticles (LNPs).

Samples	Yield [%]	EE [%]	PS [nm]	ZP [mV]	Γ_s [mg m^{-2}]
LNP[a)]	91.7	-	192 ± 4	−3.1 ± 0.3	28 ± 2
M-LNP[b)]	90.4	99.0 ± 0.6	184 ± 3	−4.6 ± 0.5	30 ± 5
NR-LNP[c)]	94.4	-	170 ± 2	−2.5 ± 0.1	30 ± 0

[a)]Blank-LNP; [b)]myricetin-loaded LNP; [c)]Nile red-loaded LNP.

Table S2. Scores (0–3) obtained from the hen's egg chorioallantoic membrane assays.

Samples	Concentration	Treated volume [mL]	Description	Score
PBS[a)]	-	2	Not irritant	0
NaOH[b)]	1 mmol mL^{-1}	2	Severely irritant	3
M[c)]	0.1 mg mL^{-1}	2	Not irritant	0
Blank-LNP[d)]	-	2	Not irritant	0
M-LNP[e)]	0.1 mg mL^{-1}	2	Not irritant	0

[a)]Phosphate buffered saline; [b)]NaOH aqueous solution; [c)]myricetin solution (5 vol% in DMSO); [d)]blank lipid nanoparticle dispersion diluted 10-fold with PBS; [e)]myricetin-loaded lipid nanoparticle dispersion diluted 10-fold with PBS.

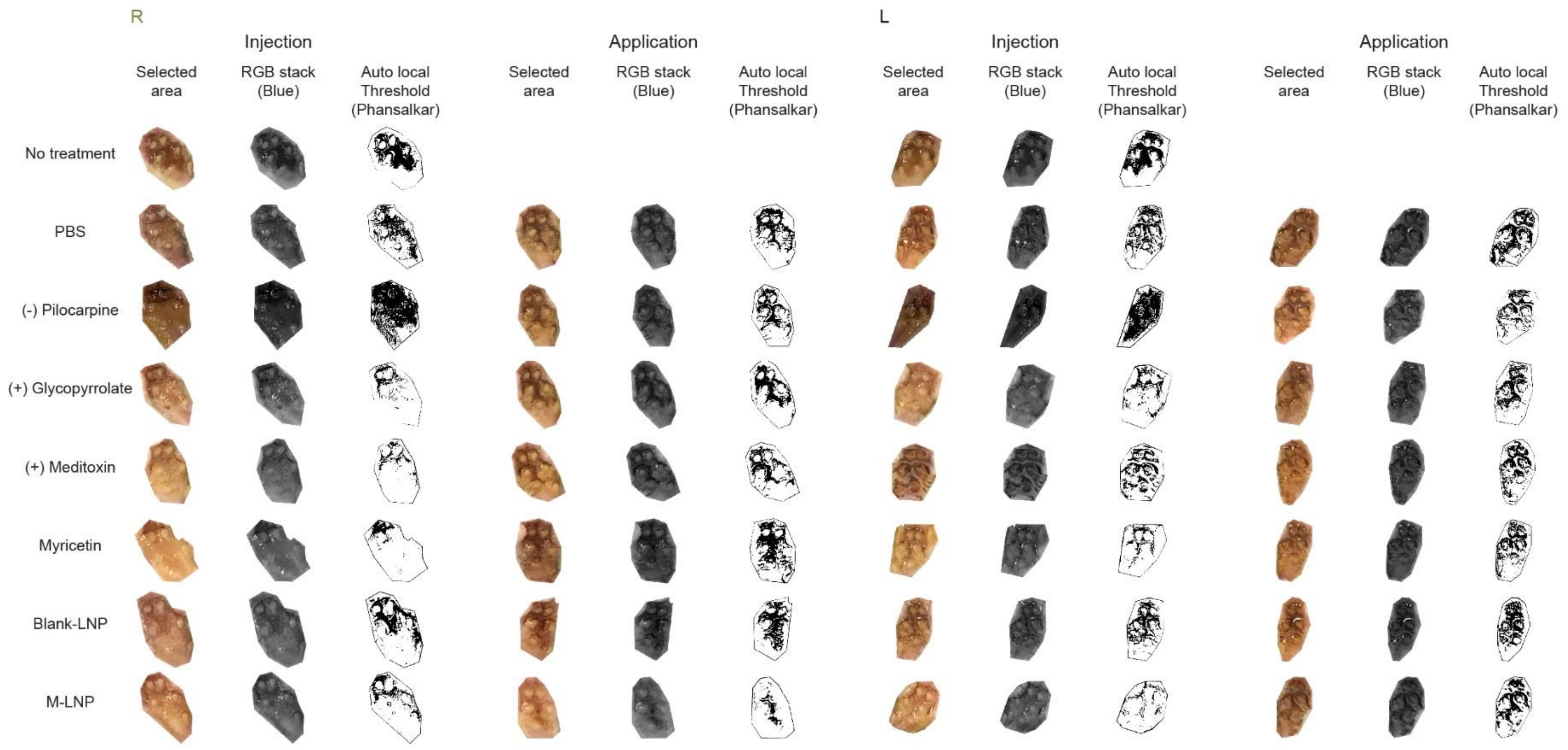

Fig. S1. Mouse hind footpad images obtained after image processing using ImageJ. Right (R) and left (L) hind footpads of mice groups sample-treated with subcutaneous injection and skin-application on the right footpads. Groups: no treatment, phosphate buffered saline (0.8 mL kg^{-1}, PBS), pilocarpine HCl (2.5 mg kg^{-1}, negative control), glycopyrrolate bromide (0.25 mg kg^{-1}, positive control), botulinum neurotoxin A type (Meditoxin; 0.8 U kg^{-1}, positive control), myricetin (M; 0.8 mg kg^{-1}), blank lipid nanoparticle (Blank-LNP; 0.8 mL kg^{-1}), and M-LNP (0.8 mg kg^{-1}).

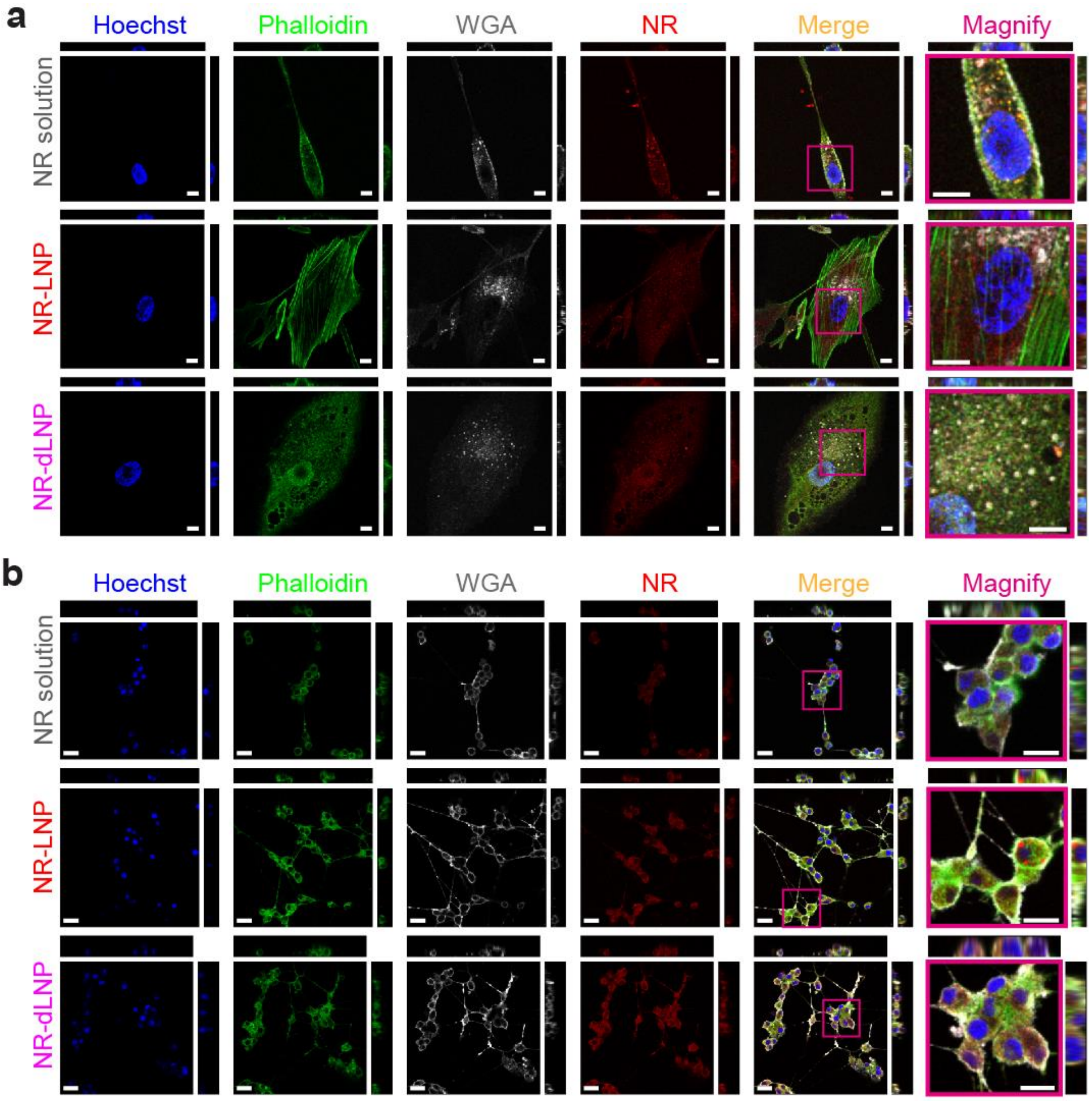

Fig. S2. Orthogonal-viewed images obtained using confocal laser fluorescence microscopy. (a) Adult human dermal fibroblasts (HDFa) and (b) differentiated PC12 cells incubated with culture media containing Nile red (NR), NR-LNP, and NR-dLNP (scale bars, 20 μm). Nuclei, filamentous actin, glycolipids/glycoproteins, and NR in cells are in blue (Hoechst, Hoechst 33258), green (Phalloidin, Oregon Green 514 phalloidin), gray (WGA, wheat germ agglutinin Alexa Fluor 647 conjugate), and red (NR), respectively. Images at the bottom-left, top-left, and bottom-right in each image are the upper, y-axis, and x-axis views, respectively.

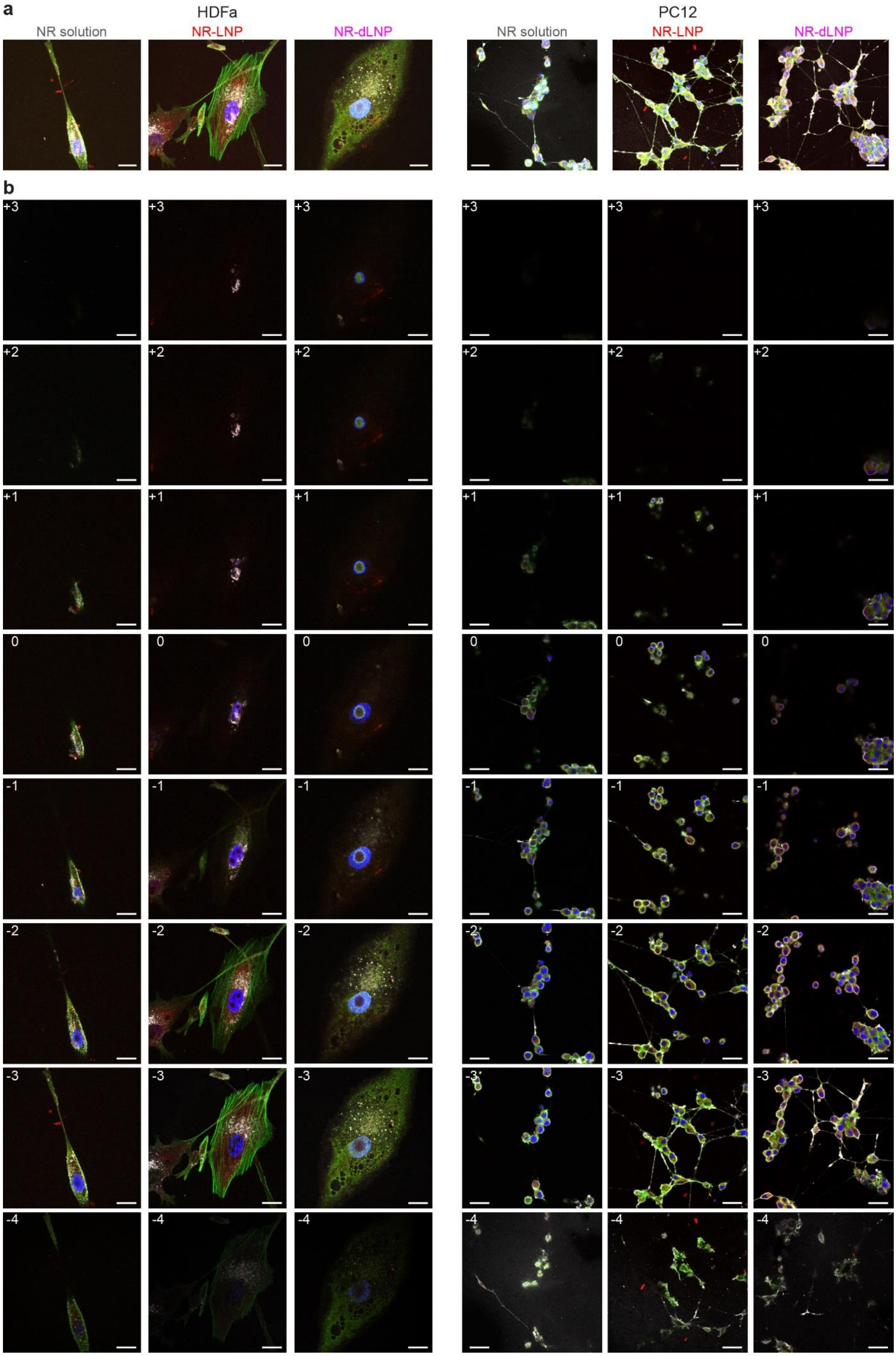

a
HDFa
NR solution
NR-LNP
NR-dLNP
PC12
NR solution
NR-LNP
NR-dLNP
b
+3
+2
+1
0
-1
-2
-3
-4

Fig. S3. (a) Upper-view and (b) z-stack images obtained using confocal laser fluorescence microscopy. Adult human dermal fibroblasts (HDFa) and differentiated PC12 cells incubated with the culture media containing Nile red (NR), NR-LNP, and NR-LNP deformed by lipolysis (NR-dLNP) (scale bars, 20 μm). Nuclei, filamentous actin, glycolipids/glycoproteins, and endocytic NR-LNPs in the cells are depicted in blue (Hoechst, Hoechst 33258), green (Phalloidin, Oregon Green 514 phalloidin), gray (WGA, wheat germ agglutinin Alexa Fluor 647 conjugate), and red (NR), respectively. Z-stack images numbered for each section obtained along the z-axis are shown with an image taken at the mid-height of the nucleus defined as zero.

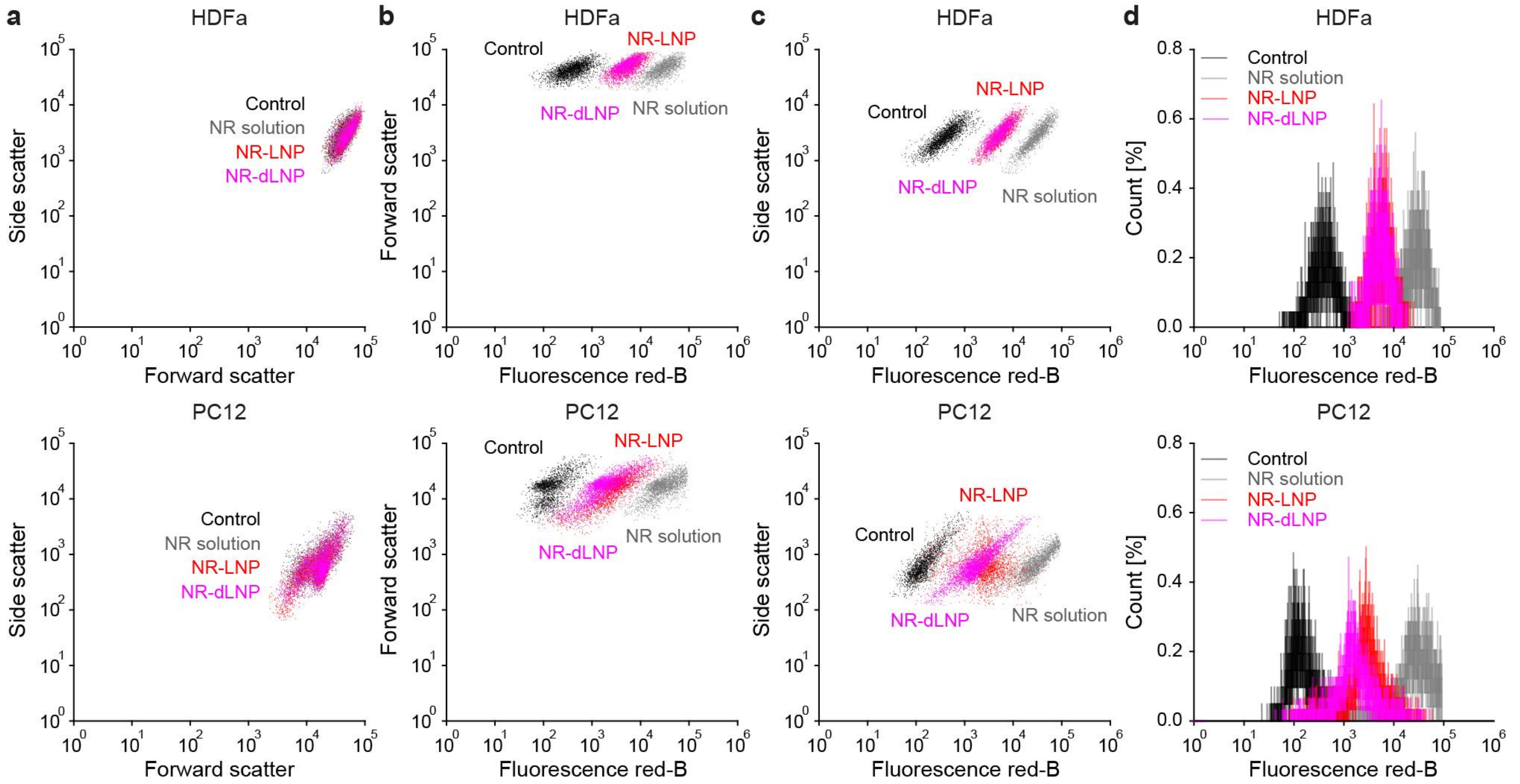

Fig. S4. Flow cytometry. Adult human dermal fibroblasts (HDFa) and PC12 cells incubated with the culture media containing Nile red (NR), NR-LNP, and NR-LNP deformed by lipolysis (NR-dLNP). Scatter plots for (a) side scatter versus forward scatter, (b) forward scatter versus fluorescence red-B, and (c) side scatter versus fluorescence red-B; (d) histograms for the fluorescence red-B signal.

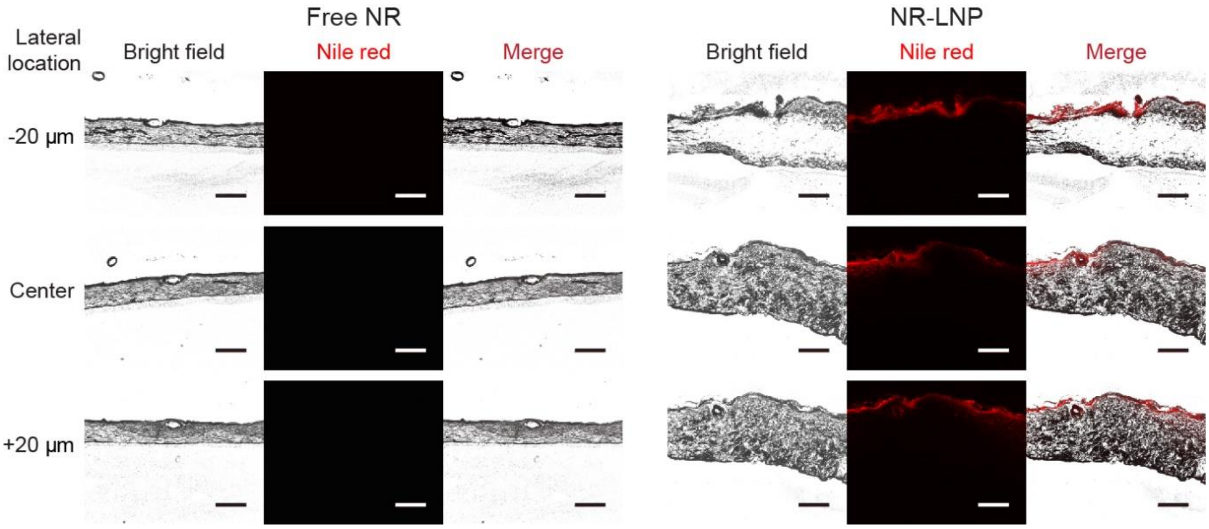

Fig. S5. Fluorescence microscopy images of porcine skin. Vertical cryo-sections after the test with NR solution (5 vol% DMSO in PBS) or NR-loaded LNP (NR-LNP) (scale bars, 500 μm). Thickness between the adjacent cryo-sections was 20 μm.
